# Severe pneumoperitoneum, mediastinal emphysema, and thoracoabdominal wall emphysema during flexible bronchoscopy: a case report

**DOI:** 10.3389/fmed.2026.1744405

**Published:** 2026-02-13

**Authors:** Jun Yang, Mei Yao, Baolong Lu, Dong Huang, Lu Yang, Guohou Zhao

**Affiliations:** Department of Respiratory and Critical Care Medicine, Northeast Yunnan Central Hospital, Zhaotong, Yunnan, China

**Keywords:** barotrauma, case report, flexible bronchoscopy, oxygen delivery method, pneumoperitoneum

## Abstract

This case presents a rare complication that occurred during flexible bronchoscopy, including severe pneumoperitoneum, mediastinal emphysema, and thoracoabdominal wall emphysema. This study provides a profound analysis of the most probable causes of the aforementioned complications, hoping to offer some valuable insights for clinical practice. The patient developed abdominal distension, abdominal bulge, decreased oxygen saturation, and hemodynamic instability during flexible bronchoscopy under intravenous anesthesia with oxygen delivery via a nasopharyngeal oxygen cannula at a flow rate of 5 L/min. Following a multidisciplinary resuscitation procedure involving the insertion of an endotracheal tube, the administration of vasoactive medications, and the performance of an abdominal paracentesis, the patient’s condition demonstrated signs of stabilisation. Imaging studies revealed pneumoperitoneum, mediastinal emphysema, and thoracoabdominal wall emphysema, but no pneumothorax was observed. Additionally, no endoscopically visible gastrointestinal perforation was detected. The patient was finally discharged after improvement with conservative treatment. This report aims to explore the potential mechanisms and management strategies for this rare complication, highlighting the need for vigilance against barotrauma and careful management of oxygen delivery methods during bronchoscopy.

## Introduction

Flexible bronchoscopy is a common interventional procedure for the diagnosis and treatment of respiratory diseases. Its frequent complications include hemorrhage, pneumothorax, mediastinal emphysema, and infection. However, the occurrence of severe pneumoperitoneum is extremely rare. Pulmonary or alveolar barotrauma may be one of its underlying mechanisms, where gas can travel through the pathway of “alveoli → lung interstitium → mediastinum → peritoneal cavity,” causing mediastinal emphysema and pneumoperitoneum. Meanwhile, when there is occult gastrointestinal injury, this complication may also occur. For instance, in cases of esophageal injury, gas can pass through the pathway of “esophagus → mediastinum → abdominal cavity,” leading to mediastinal emphysema and pneumoperitoneum. Similarly, in cases of gastrointestinal injury, gas can travel through the pathway of “gastrointestinal tract → abdominal cavity → mediastinum,” resulting in pneumoperitoneum and mediastinal emphysema (1, 2). The nasopharyngeal oxygen cannula is a commonly employed method for oxygen delivery during bronchoscopy. This report details a case of sudden mediastinal emphysema and severe pneumoperitoneum that occurred during a flexible bronchoscopy while the patient was receiving oxygen via a nasopharyngeal oxygen cannula. Reviewing in conjunction with the literature, this case study aims to enhance our awareness of preventing this complication and our ability to respond to it.

## Case presentation

The patient, a 68-year-old male farmer, was admitted to the hospital with a history of “cough, expectoration, shortness of breath, and fever for over one week.” The cough was paroxysmal, with copious white sticky sputum production. Shortness of breath was more pronounced during activity and slightly relieved with rest. No blood in sputum or hemoptysis was observed, nor was there pink frothy sputum. The patient reported subjective fever, though no external temperature measurement was taken. No chills or shivering were noted, and there was no significant weight change since the onset of symptoms. The patient had a nine-year history of sleep apnea-hypopnea syndrome. There was no history of chronic pulmonary or cardiac disease, thoracic surgery, immunosuppressive disorders, or use of immunosuppressive medications. The patient denied any history of smoking, alcohol consumption, or familial hereditary diseases. Physical examination upon admission revealed a body temperature of 38.1 °C, a pulse rate of 90 beats per minute, a respiratory rate of 20 breaths per minute, a blood pressure of 124/68 mmHg, and an oxygen saturation of 91% on room air. The patient’s body mass index was 31.6. The patient was alert and oriented, with no cyanosis observed in the lips or extremities. Bilateral breath sounds were diminished, and moist rales were audible in the lower lung fields bilaterally, with no dry rales detected. Basic laboratory findings indicated the following: blood cell analysis: total white blood cell count 12.18 × 10⁹/L, absolute neutrophil count 9.94 × 10⁹/L, absolute lymphocyte count 1.34 × 10⁹/L, absolute eosinophil count 0.02 × 10⁹/L, C-reactive protein (CRP) 204.50 mg/L, procalcitonin (PCT) 0.38 ng/mL, fibrinogen (FIB) 8.01 g/L, liver function, renal function, electrolyte, brain natriuretic peptide (BNP) were in the normal range. Arterial blood gas analysis: PH 7.43, PaCO2 36 mmHg, PaO2 61 mmHg, HCO3–23.90 mmol/L. The chest CT scan revealed multiple ground-glass opacities, patchy opacities, and nodular shadows in both lungs. Preliminary diagnostic consideration: pulmonary infection. To obtain further microbial samples from the lower respiratory tract and exclude non-infectious diseases such as interstitial lung disease and malignant tumors, a bronchoscopy under general anesthesia was planned after excluding relevant contraindications. Preoperative local anesthesia was administered with 2% lidocaine nasal spray, and a nasopharyngeal oxygen cannula was inserted for oxygen delivery. The depth of the catheter tip was approximately 5 cm from the nostril, with the final position expected to reach the nasopharynx. The oxygen flow rate was set at 5 L/min. Prior to the procedure, the patient received intravenous administration of 10ug sufentanil citrate injection and 0.1 g propofol injection. After the anesthesia took effect, the patient’s oral cavity remained closed without any signs of airway obstruction.

**Figure 1:**
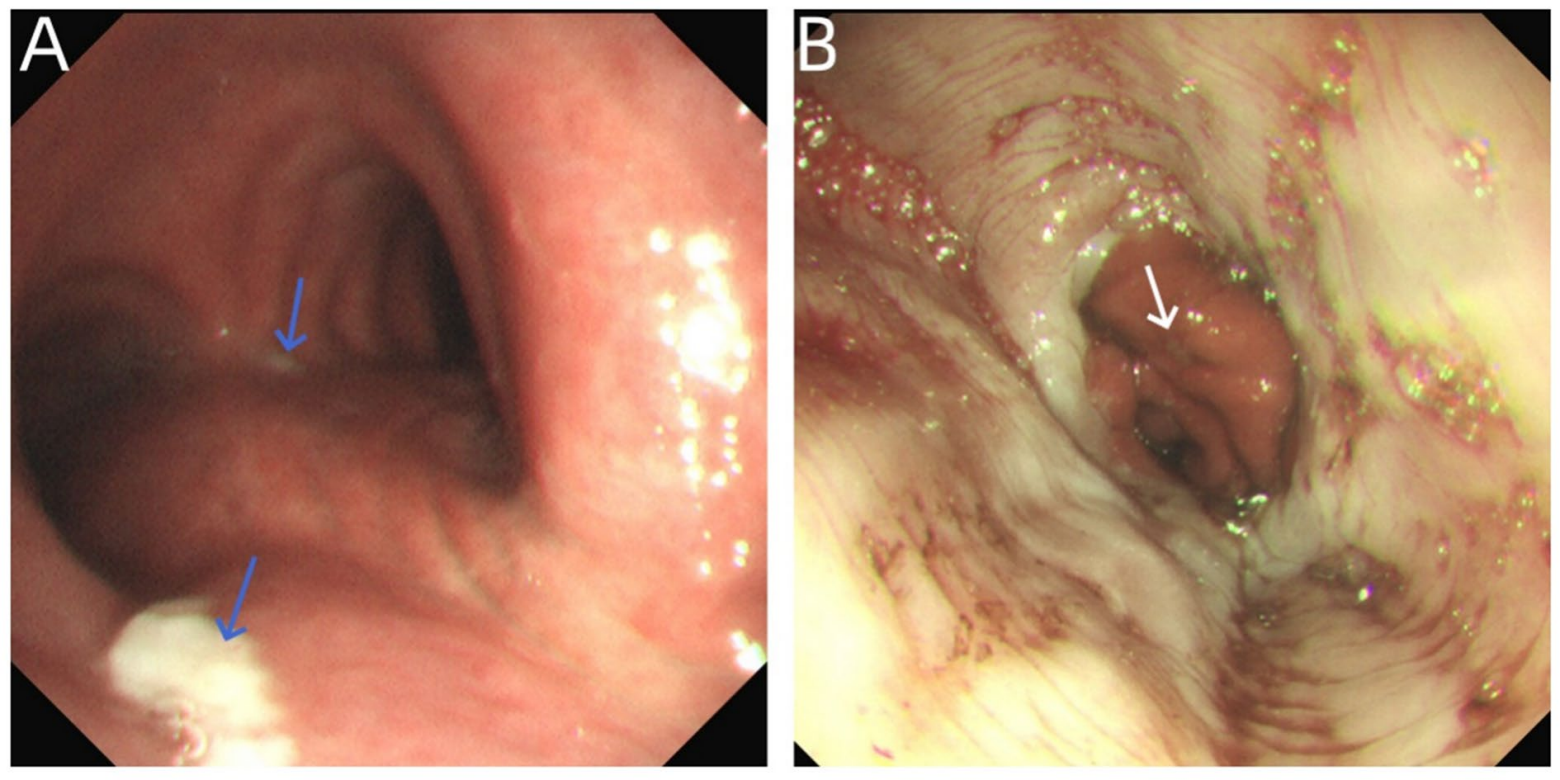
**(A)** Bronchoscopy: Endoscopic examination revealed significant congestion of the tracheal and bronchial mucosa. The wall of the lower trachea and the right main bronchus was covered with a little white material (shown by the blue arrows). The carina was sharp. The bilateral main bronchi, lobar bronchi, and segmental bronchi were patent, with smooth and intact mucosa, and no damage or perforation of the bronchial walls was observed. **(B)** Diagnostic gastroduodenoscopy: Endoscopic findings showed smooth esophageal mucosa with a superficial ulcer measuring approximately 1 × 2.5 mm at the cardia, but no perforation and bubble overflow (shown in white arrow) were observed. No perforation, ulcer, or space-occupying lesions were observed in the gastric fundus, gastric body, gastric angle, gastric antrum, and duodenum, and a little gas was accumulated in the stomach and duodenum.

**Figure 2:**
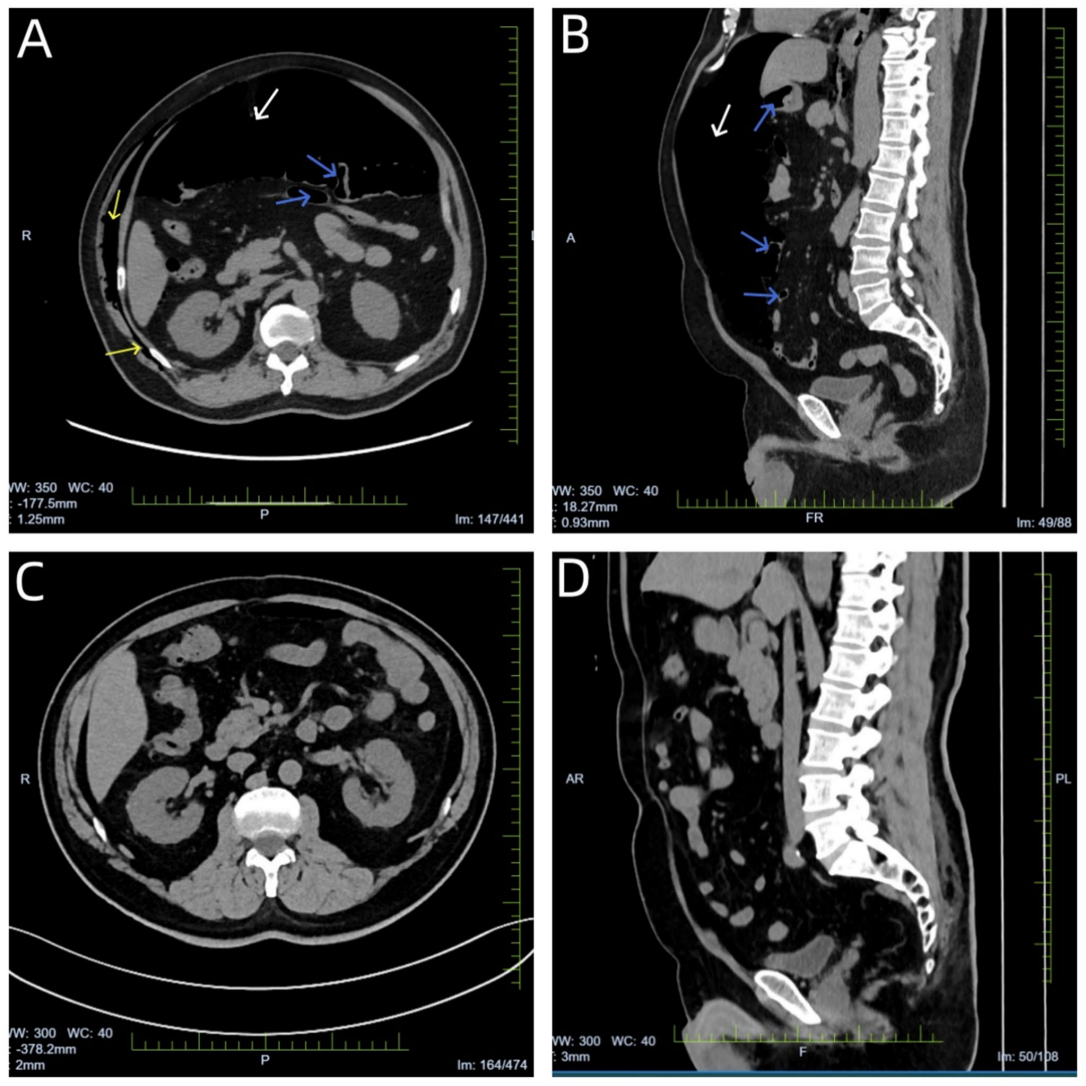
**(A,B)** CT scan performed on the day of emergency treatment revealed massive free gas in the abdominal cavity (shown by white arrow), multiple gas accumulations in the right thoracoabdominal wall and muscle compartments (shown by yellow arrows), slight gas accumulation in the jejunum with mild luminal dilatation (shown by blue arrows). **(C,D)** Follow-up abdominal CT on day 6 showed near-complete absorption of free gas in the abdominal cavity and multiple gas accumulations in the thoracoabdominal wall and muscle compartments; no gas was observed within the intestinal lumen.

The endoscope was introduced through the nasal cavity at 12:45. Lidocaine was sprayed locally at the glottis and within the airway for anesthesia. Endoscopic examination revealed significant mucosal hyperemia in the trachea and bronchi. A small amount of white material was observed covering the wall of the distal trachea and the right main bronchus. The bilateral main bronchi, lobar bronchi, and segmental bronchi were patent, with smooth and intact mucosa. No evidence of wall injury or perforation was noted (Figure 1A). Following endoscopic entry, a simple bronchoalveolar lavage was performed only in the apical segment and posterior segment of the right upper lobe and the basal segments of both lower lobes. The lavage fluid and sputum were gently aspirated. No brushing, biopsy, or puncture procedures were conducted. During the procedure, no severe coughing, Valsalva-like maneuvers, patient movement, bronchospasm, increased resistance, intubation difficulty, or mask positive pressure ventilation occurred. Several minutes into the procedure, the bronchoscope reached the right lower lobe basal segment for lavage, and abdominal bulge was observed in the patient. Electrocardiogram (ECG) monitoring revealed a heart rate of 108 beats per minute, oxygen saturation levels of 87%, blood pressure of 98/55 mmHg, and a respiratory rate of 23 breaths per minute. Physical examination revealed mild cyanosis of the lips, abdominal bulge, and full abdominal percussion drum sound. The bronchoscopy was promptly discontinued, and urgent consultations were initiated from the departments of gastroenterology, gastrointestinal surgery, and anesthesiology. The nasopharyngeal oxygen cannula was promptly removed and replaced with a face mask for the purpose of oxygen therapy. The patient’s condition exhibited a marked progression of deterioration. Two minutes later, the patient’s heart rate decreased to 50 beats per minute (bpm), the oxygen saturation dropped to 55%, the respiratory rate increased to 30 breaths per minute, and the blood pressure fell to 90/50 mmHg. The patient underwent immediate tracheal intubation, and mechanical ventilation was initiated. The ventilator was configured in pressure control ventilation (PCV) mode with the following parameters: a fraction of inspired oxygen of 60%, an inspiratory pressure of 12 cmH2O, a respiratory rate of 18 breaths per minute, an inspiratory to expiratory time ratio of 1:2, and a positive end-expiratory pressure of 4 cmH2O. Under this configuration, the tidal volume ranged from 400 to 450 mL. Concurrently, 1 mL of epinephrine hydrochloride be administered intravenously via a bolus injection. Subsequently, vital signs exhibited improvement, with a heart rate of 105 beats per minute, oxygen saturation levels of 90%, blood pressure of 100/60 mmHg. Due to the presence of marked abdominal distension, the patient was given a bedside diagnostic gastroduodenal endoscopy and assisted exhaust. Endoscopic findings revealed smooth esophageal mucosa and a superficial ulcer measuring approximately 1 × 2.5 mm at the cardia, but no perforation or bubble overflow. No perforations, ulcers, or space-occupying lesions were detected in the gastric fundus, body, angle, antrum, or duodenum. A small amount of gas was present in the stomach and duodenum (Figure 1B). Consequently, the abdominal distension was primarily attributed to pneumoperitoneum. Right abdominal paracentesis was performed immediately using a 10-ml syringe. Following the procedure, a large number of bubbles overflowed, and the abdominal distension progressively resolved. Thereafter, the patient’s vital signs gradually stabilized, and his consciousness returned. The endotracheal tube was removed and the patient transitioned to mask-assisted ventilation with a noninvasive ventilator. Thirty minutes later, a marked decrease in the number of bubbles in the syringe was observed, and the patient reported significant relief from abdominal distension. The syringe was then removed, and the oxygen delivery method was switched to face mask oxygen therapy (rescue timeline shown in Table 1). After a few minutes, the patient underwent emergency chest and abdominal CT scans under the supervision of medical personnel (Figures 2A,B). The scans revealed minor gas accumulation in the mediastinum, multiple gas collections in the right thoracoabdominal wall and muscle compartments, substantial free gas remaining in the abdominal cavity, and minor gas accumulation in the jejunum with slight luminal dilatation.

**Table 1:** Rescue timeline on the day of bronchoscopy.

Time point	Change in the patient’s condition	Treatment measures
12:45	Vital signs stable, no contraindications.	After anesthesia took effect, proceeded with endoscopic examination via the nose. No severe coughing, Valsalva maneuver, or restlessness during the procedure.
12:50	The patient was found to have an abdominal bulge, with a HR 108 bpm, SpO2 87%, BP 98/55 mmHg, and R 23 bpm. Physical examination revealed mild cyanosis of the lips, abdominal bulge, and whole abdominal percussion drum sound.	Discontinued bronchoscopy. Requested multidisciplinary assistance for emergency treatment. Removed the nasopharyngeal oxygen cannula and replaced it with mask oxygen delivery.
12:52	HR decreased to 50 bpm, SpO2 55%, R 30 bpm, and BP 90/50 mmHg.	Tracheal intubation was followed by anesthesia ventilator-assisted breathing, and 1 ml of adrenaline hydrochloride was injected intravenously.
12:55	Vital signs improved: HR 105 bpm, SpO2 90%, BP 100/60 mmHg, R 22 bpm. However, marked abdominal distension persists.	Bedside diagnostic gastroduodenoscopy with assisted degassing was performed. However, no signs of perforation were observed.
12:58	The abdominal distension was primarily attributed to pneumoperitoneum.	Performed a right upper quadrant abdominal paracentesis with a syringe to release gas.
13:08	Vital signs were stable. The patient was alert. Abdominal distension was gradually resolving.	The tracheal tube was pulled out and adjusted to non-invasive ventilator assisted ventilation.
13:20	Abdominal distension was significantly reduced, and the number of bubbles escaping from the syringe was markedly decreased.	The syringe was pulled out, and the non-invasive ventilation was replaced by a mask for oxygen delivery.

HR: Heart rate, BP: Blood pressure, R: Respiration, SpO2: oxygen saturation.

Subsequent to a multidisciplinary conference involving Respiratory and Critical Care Medicine, Gastroenterology, Gastrointestinal Surgery, and Anesthesiology, the comprehensive opinion was as follows: The origin of the gas present in the mediastinum, abdominal cavity, and intestinal lumen remained to be elucidated. A diagnostic gastroduodenoscopy was performed, which revealed no obvious perforation. In light of the patient’s current improvement in symptoms and the absence of indications for an emergency exploratory laparotomy, we recommend symptomatic supportive treatment, including fasting, gastrointestinal decompression, and anti-infective therapy. After communicating with the patient’s family, the operation of exploratory laparotomy was not considered. A follow-up abdominal CT scan, conducted six days after the commencement of treatment—which included two days with a nasogastric tube, three days of fasting, and supportive care—demonstrated near-complete resolution of pneumoperitoneum (Figures 2C,D). Targeted Next-generation Sequencing (tNGS) of bronchoalveolar lavage fluid identified suspected pathogenic microorganisms, including *Mycoplasma pneumoniae*, *Haemophilus influenzae*, and Influenza A virus (H1N1). The pulmonary infection improved after two weeks of antibiotic therapy, and the patient was subsequently discharged. Three weeks later, the patient was contacted by telephone and advised to return to the hospital for a follow-up examination. However, the patient said that he was in good condition and refused to return to the hospital for review.

## Discussion

**Figure 3:**
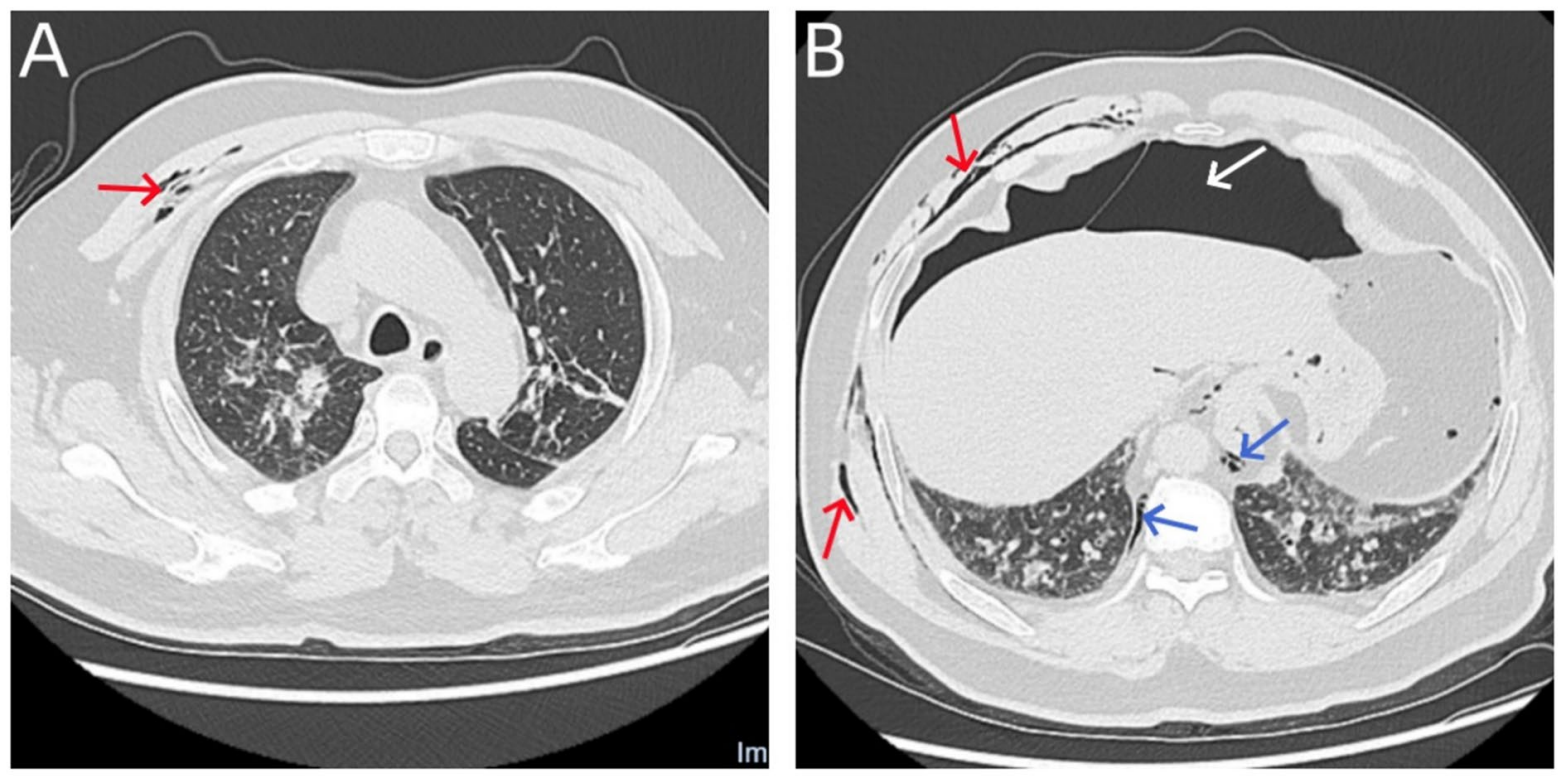
**(A,B)** Chest CT scan performed on the day of emergency treatment revealed multiple air collections in the right thoracoabdominal wall and muscle compartments (shown by red arrows), minimal air accumulation in the mediastinum (shown by blue arrows), and a large amount of free gas in the abdominal cavity (shown by white arrow). However, no signs of pneumothorax were observed.

The incidence of bronchoscopy complications varies across different literature sources, but the rate of severe complications is generally below 1% (3, 4). In this case, bronchoscopy was performed with oxygen delivery via a nasopharyngeal oxygen cannula inserted through the nostril. During the procedure, abdominal bulge and a decline in oxygen saturation were observed, prompting immediate and active resuscitation. Suspecting pneumoperitoneum, abdominal paracentesis was performed for decompression. Subsequent imaging revealed pneumoperitoneum, mediastinal emphysema, and subcutaneous emphysema of the thoracoabdominal wall. Notably, pneumothorax was absent, which constitutes a rare complication (Figures 3A,B).

The following potential mechanisms may be considered: Initially, The existence of mediastinal-abdominal gas diffusion pathways. The “McKinley effect” illustrates that during instances of severe coughing, alveoli may undergo rupture due to heightened pressure, thereby enabling air to traverse the bronchovascular sheath into the mediastinum and further access the abdominal cavity through the diaphragmatic hiatus (5, 6). However, no severe cough or respiratory distress occurred during the operation on the patient. At the same time, pneumothorax was very likely to occur if alveolar rupture occurs, but the patient had no pneumothorax, so the possibility of this mechanism is extremely low. In addition, the patient may had airway weakness, bronchoscopy may caused minor damage to the airway, and the gas diffused along the tissue space to the mediastinum, abdominal cavity, and thoracoabdominal wall (7). This patient cannot excluded the possibility of this mechanism. Finally, the improper position of the nasopharyngeal oxygen cannula may increased the risk of gas entering the esophagus and stomach. The pharyngeal-esophageal one-way valve mechanism explains the possibility. After the gas entered the digestive tract, the pressure of the digestive tract increased and a small perforation occured, but it was not found during gastro-duodenoscopy. Due to fecal filling in the intestinal tract, the patient was in a supine position, allowing gas to ascend into the abdominal cavity and mediastinum upward, resulting in pneumoperitoneum, mediastinal emphysema, and thoracoabdominal wall emphysema. The increased intra-abdominal pressure may compressed the intestinal space, leading to a phenomenon where a large volume of gas accumulated in the abdominal cavity while only a small amount remained in the intestinal lumen. This patient was more likely to have the above complications through the last mechanism.

There are many related literatures on bronchoscopy complicated with pneumothorax or mediastinal emphysema, but less complicated with pneumoperitoneum. By reviewing 12 patients with pneumoperitoneum during bronchoscopy in PubMed, Medline, Web of Science, Cochrane Library and other databases in the past 30 years, 9 of them were flexible bronchoscopy, and the rest were 2 cases of rigid bronchoscope and 1 case of robotic bronchoscopy. Oxygen delivery via nasopharyngeal oxygen cannula accounted for 66% (8 cases), and flexible bronchoscopy was performed in all 8 patients. Among them, 3 underwent Transbronchial Lung Biopsy (TBLB) or Transbronchial Needle Aspiration (TBNA), 1 underwent brush biopsy, and the remaining 4 underwent only observational procedures. Of these, 5 cases were complicated by pneumothorax and/or mediastinal emphysema (including 1 TBLB, 1 TBNA, 1 brush biopsy, and 2 observational procedures), while the other 3 cases presented with isolated pneumoperitoneum (including 1 TBNA and 2 observational procedures) (6, 8–10). These data indicate that the method of oxygen delivery via a nasopharyngeal oxygen cannula during bronchoscopy has the highest likelihood of inducing pneumoperitoneum. This phenomenon may be attributed to the decline in pharyngeal muscle strength that occurs during anesthesia, and the front end of the nasopharyngeal oxygen cannula is too close to the pharynx. As the inspiratory pressure in the esophagus increases, a one-way valve mechanism is formed, allowing gas to enter the digestive tract (9). Consequently, when circumstances allow, the utilisation of nasopharyngeal oxygen cannula should be eschewed wherever practicable. It is recommended that alternative methods be considered, such as the mask oxygen delivery. If nasopharyngeal oxygen cannula is used, its placement position should be accurately grasped, and close monitoring of abdominal signs and respiratory movements is essential, and emergency equipment must be kept readily available.

The diagnosis in this case was based on clinical presentation and imaging studies. The presence of bubbles observed during abdominal paracentesis further confirmed pneumoperitoneum. During the resuscitation efforts, there was a progressive decrease in oxygen levels, which required the immediate insertion of an endotracheal tube by the anesthesiology team. A subsequent gastroscopy revealed no significant perforative lesions in the esophagus, stomach, or duodenum. Multidisciplinary collaboration is of particular importance in the management of such complex cases. A conservative management strategy was selected. In the absence of peritonitis signs, no abnormal findings on gastroscopy, and rapid stabilization of vital signs, surgical intervention was not pursued after consultation with the patient’s family (8).

## Conclusion

**Figure 4:**
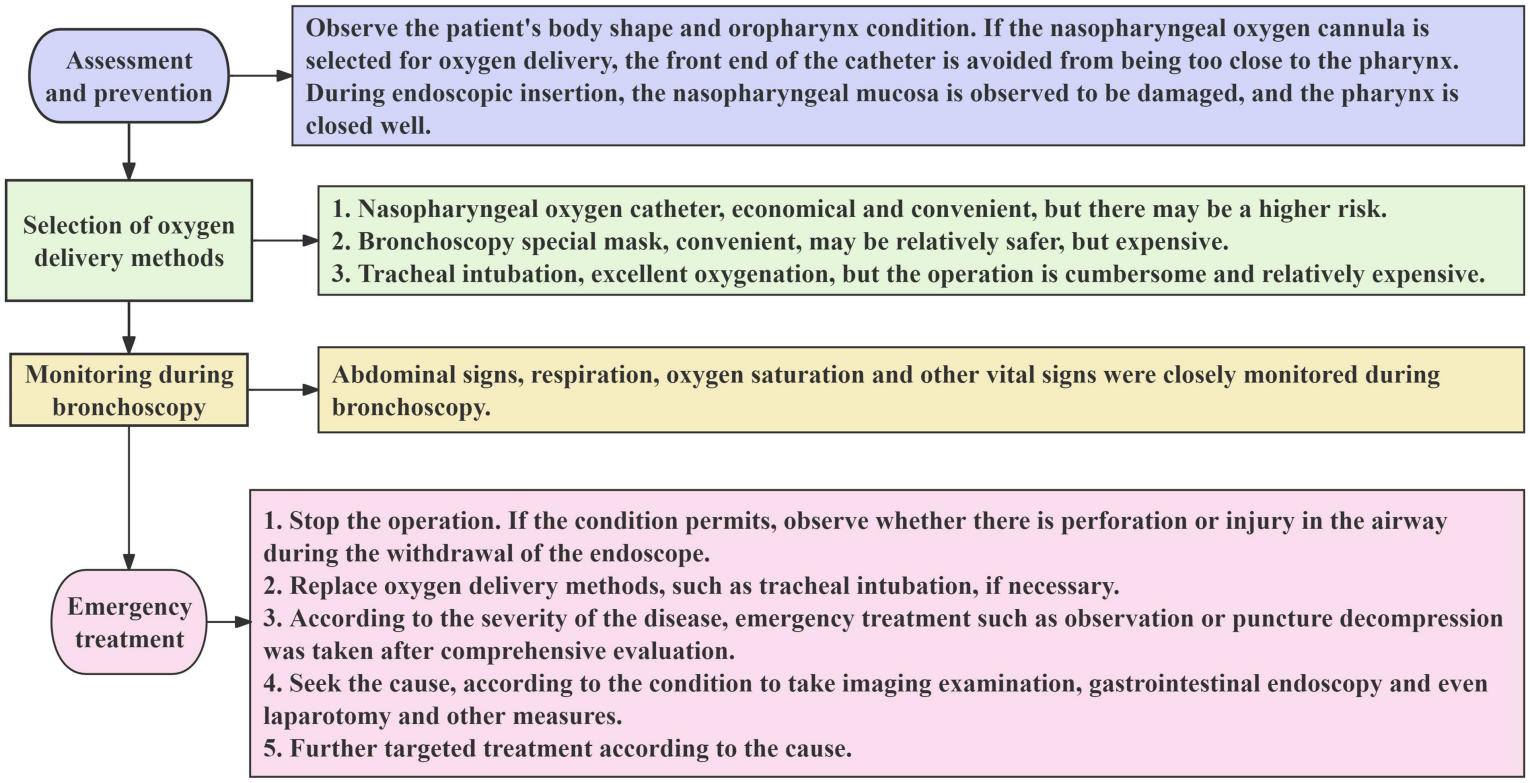
Brief prevention and treatment process of bronchoscopy complicated with pneumoperitoneum.

During bronchoscopy, severe pneumoperitoneum, mediastinal emphysema, and thoracoabdominal wall emphysema occurred without the development of pneumothorax, which is a rare complication. There may be a greater potential risk of oxygen delivery by a nasopharyngeal oxygen cannula, especially when the tip of the catheter is too close to the pharynx. Sometimes, a small detail may cause a huge clinical disaster. Clinicians should be vigilant, conduct thorough preoperative evaluations and preventive measures, choose a safer oxygen delivery strategy, and strengthen intraoperative monitoring and emergency preparedness (Figure 4). If patients with such complications have no acute peritonitis, obvious perforation signs and receive close multidisciplinary monitoring, conservative treatment such as abdominal puncture may be successful, unnecessary surgical intervention may be avoided, and the recovery time of patients may be shortened to a certain extent, and the prognosis of patients may be improved.

Finally, although we made a timely judgment on the condition at the critical moment, and the multidisciplinary team worked together to make the right response, avoiding the rupture of the abdominal cavity due to high pressure and saving the patient’s life. However, we also have some areas for improvement. The patient’s pneumoperitoneum was likely to occur earlier than the time we found. The patient’s abdomen was covered with a small quilt to keep warm. When the early abdominal bulge was not obvious, it was not found earlier. In the future, we should always pay attention to the patient’s abdomen during bronchoscopy. Due to the emergency situation, there is no picture of the patient’s abdominal bulge and puncture deflation, which is a small regret. Additionally, the patient’s pharyngeal closure was not carefully examined during bronchoscopy withdrawal, and a pneumoperitoneum puncture kit was not prepared in advance, with a syringe being used instead. These may provide some lessons for similar clinical work. Regrettably, the aforementioned complications in this patient were inferred through clinical manifestations and imaging studies. The patient’s condition rapidly improved, and after multidisciplinary consultation and comprehensive consideration of the patient’s and family’s opinions, exploratory laparotomy was not performed. This inference ultimately could not be definitively substantiated.

## Patient perspective

I was unfortunate to experience this severe complication during the course of treatment, but in this extremely critical moment, I was also lucky to have been successfully rescued by the medical staff. The process of treatment and rehabilitation was painful for me, but I can also understand the individual differences in medical activities and some risks that cannot be absolutely controlled. I am relieved to have achieved a successful recovery.

## Data Availability

The original contributions presented in the study are included in the article/supplementary material, further inquiries can be directed to the corresponding author.
